# Using community-based participatory research in improving the management of hypertension in communities: A scoping review

**DOI:** 10.4102/safp.v62i1.5039

**Published:** 2020-07-16

**Authors:** Pugie T. Chimberengwa, Mergan Naidoo

**Affiliations:** 1 Discipline of Public Health Medicine, School of Nursing and Public Health, University of KwaZulu-Natal, Durban, South Africa; 2 Ministry of Health and Child Care, Zimbabwe; 3 Discipline of Family Medicine, School of Nursing and Public Health, University of KwaZulu-Natal, Durban, South Africa

**Keywords:** Hypertension, community-based participatory research, prevention and control, participatory action research, health and social care

## Abstract

**Background:**

Hypertension (HT) is a key contributor to cardiovascular diseases (CVDs). The improved management of HT in the community and primary care settings should be a priority for low- and middle-income countries (LMICs). Improving the prevention and management of HT in primary care settings should also be a priority for developing countries. There is a need for more studies using community-based approaches that show the impact of these programmes on HT outcomes, which may motivate policymakers to invest in such approaches. The ward-based outreach team or village healthcare worker models were meant to provide such approaches, but many of these have become lower levels of curative care. We conducted a scoping review to examine how community-based participatory research (CBPR) was being used to improve HT management.

**Methods:**

Several electronic databases were searched, namely PubMed, MEDLINE, Google Scholar and Web of Science, generating 798 references. The publications were screened through several rounds. Data were extracted and imported into a Microsoft Excel spreadsheet, numerically summarised and qualitatively analysed.

**Results:**

Nine articles were included. These publications originated from the United States, Colombia, Canada, China, South Africa and Zimbabwe. Mixed methods, qualitative, randomised control trials and quasi-experimental studies were used to implement CBPR in the studies included. All the studies addressed complex health problems and inequities among the minorities utilising multiple stakeholder participation. Academic–community coalitions were formed, which enabled engagement and sharing of power equitably. As a result, there was acceptability and sustainability of interventions.

**Conclusion:**

A CBPR framework can be used to define the context, group dynamics, implementation and outcomes of HT. It is possible to apply CBPR in HT management to appropriately address health disparities while emphasising a community-driven approach. To achieve this, tailored health education platforms should be developed and implemented.

## Background

Hypertension (HT) is a global public health condition and a leading cause of death and disability in developing countries.^1^ The prevalence of HT is highest in the African region at 46% of adults aged 25 years and above, while the lowest prevalence of 35% is found in the Americas.^1^ Not only is HT more prevalent in low- and middle-income countries (LMICs) as a result of weak health systems, but the number of people who are undiagnosed, untreated and uncontrolled is also high.^1,2^ Hypertension rarely causes symptoms in the early stages and many people go undiagnosed.^2^ Those who are diagnosed may lack access to care and treatment and may fail to control their illness over the long-term.

Higher incidence rates for HT are attributed to aging, unhealthy food intake, living a sedentary lifestyle, smoking, excessive alcohol intake, physical inactivity and chronic stress.^3^ The poor management of chronic diseases at primary care level results in a huge burden in treating complications at secondary care and results in high morbidity, premature death and subsequent premature loss of human capital.^4^ Improving the management of chronic diseases in primary care settings should be a priority for LMICs.^5^ The predominant medical model for intervention in the management of HT adopts a top-down approach where policies and management decisions are made at the macro level to be implemented at the micro level.^6^

There is a need for more studies using community-based approaches that show the impact of these programmes on HT outcomes, which may motivate policymakers to invest in such approaches. The ward-based outreach team or village healthcare worker models were meant to provide such approaches, but many of these have become lower levels of curative care.^7,8^ It is proposed that the most cost-effective and sustainable strategy is community-based participatory research (CBPR).^7,8^ A community is defined as a unit of identity, solution and practice.^9,10^ A CBPR is a relatively new approach and several terms such as ‘participatory action research’, ‘participatory research’ or ‘action research’ have been loosely and interchangeably used in literature. Community-based participatory research is a viable approach to collaborative research and improving community health.^10,11^ The CBPR framework emphasises equal partnership of expertise and decision-making, and ownership of the research between the community and the academic researchers. Furthermore, it appropriately addresses health disparities while emphasising a community-driven approach. It also supports capacity building, and the implementation and dissemination of research, and facilitates the sustainability of programmes in the community.^7,12^

The health outcomes for the management of HT are both subjective and objective in that there are many truths, hence the adoption of a mixed methods approach. To understand HT outcomes, one needs to delve into the subjective and contextual issues in the community and appreciate the importance of evidence-based medicine. The CBPR approach seeks to address these challenges as compared to a purely biomedical approach.^8,11,12^ Therefore, the interactive CBPR model^13^ outlines at a system level capacities that are developed, policies and practices that are improved and sustained interventions that were used. It takes into consideration the context of where the research is being done, group dynamics, equitable partnerships that can be formed, the intervention and the outcomes.^12^ The outcomes reflect on system and capacity changes with regards to policies or practices, sustained interventions, changes in power relations and cultural renewal.^12,13^ These will have an effect on improved health by reducing disparities while social justice is achieved.

There is a small but increasing body of literature that describes the use of CBPR in the prevention and control of HT. However, the uses of the CBPR approach in HT care seeks to understand the experiences of communities, including those patients living with HT. The standard HT prevention practices that are in place have shown limitations as the burden of HT is increasing. The increase in HT prevalence may signify that there are problems in both primary and secondary prevention strategies which have been in use thus far.^6^ We undertook a scoping review of existing literature on how CBPR has been applied in communities to improve HT management. The intention was to improve our knowledge base for the wider CBPR project on HT in a rural, disadvantaged community.

## Methods

Scoping studies are an increasingly popular approach to reviewing evidence in health research.^14,15^ Researchers may conduct scoping reviews instead of systematic reviews where the purpose of the review is to identify knowledge gaps, scope a body of literature, clarify concepts or investigate research conduct.^16^ Typically, these reviews provide a descriptive narrative that represent a synthesis of primary evidence available. The scoping study was part of a bigger project that had ethical approval obtained jointly from the Medical Research Council of Zimbabwe and the Biomedical Research Council of the University of KwaZulu-Natal, South Africa.

**FIGURE 1:**
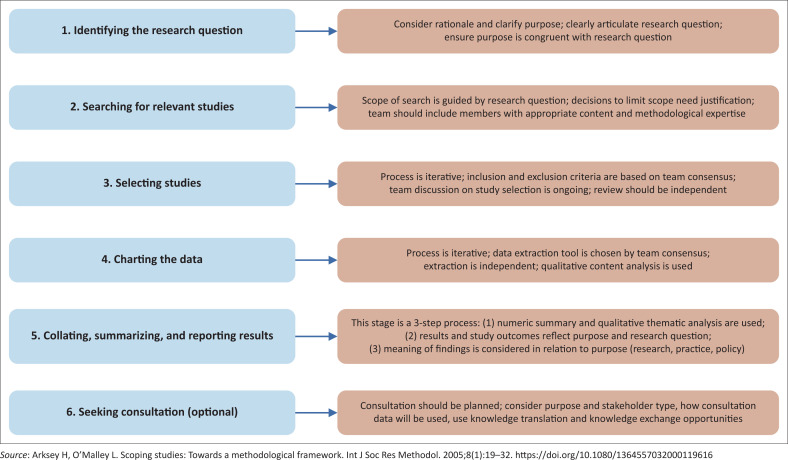
Scoping review stages as developed by Arksey and O’Malley.

We aligned our methods to the first five of the six stages of the framework proposed by Arksey and O’Malley, and Levac et al., as shown in Figure 1.^15,17^

### Stages 1 and 2

Our team comprised of a P.C. (principal investigator) and M.N. (supervisor), who agreed on the purpose of the study and a plan to guide the review. The specific research questions of the review were as follows: (1) What methodologies have been used in implementing CBPR to prevent HT? (2) How have partnerships developed through the CBPR approach? (3) What were the results of implementing CBPR to manage HT? (4) What gaps were identified when CBPR was used to manage HT?

### Criteria for including or excluding studies (stage 3)

The inclusion criteria for this scoping review included:

- Peer-reviewed manuscripts published in journals
- Articles that have been published in the English language (due to limitations in translation capacity)
- Both quantitative and qualitative methodologies
- Studies that have mentioned CBPR in HT management

All articles searches were begun in December 2017, with the last search being done in January 2020. We included all peer-reviewed publications regardless of the year of publication. There was a limit based on language, while the site was limited to the databases mentioned below.

The following electronic databases were searched: PubMed, MEDLINE, Google Scholar and Web of Science. Each database was searched for relevant titles and abstracts. Medical subject headings (MeSH) terms included ‘community based participatory research’, ‘hypertension action research’, ‘hypertension’, ‘community hypertension awareness’, ‘participatory action research’, ‘action research’, ‘community hypertension management’, ‘community blood pressure management’ and ‘hypertension implementation research’. Both authors agreed that these terms were appropriate to enable a comprehensive search for the scoping review. The search strategy shown in Box 1 generated a total of 798 references. The reference lists of the retrieved articles were cross-referenced in order to identify additional relevant papers.

BOX 1 Search strategy for hypertension prevention and control, and community-based participatory research literature.

1. ‘community based participatory research’ [MeSH] or ‘hypertension action research’ or ‘hypertension’ or ‘community hypertension awareness’ or ‘participatory action research’ or ‘action research’ or ‘community hypertension management’
2. ‘action research’ or ‘participatory research’ or ‘hypertension implementation research’ or ‘blood pressure’ or ‘community blood pressure management’
3. 1 and 2
4. ‘Community based’ or ‘hypertension’ or ‘community hypertension’ or ‘participatory action research’
5. 3 and 4

### Study selection, data collection and interpretation (stages 4 and 5)

Using the pre-specified eligibility criteria, the first round of reviews created a shortlist by screening each publication’s title and abstract for appropriateness. In this way, articles that were not in the scope of the review were eliminated. The shortlist was made by the P.C., and the M.N. reviewed the selection. Full articles were obtained and reviewed by the P.C. and M.N. Disagreements between authors were resolved by a discussion leading to mutual consent.

A data charting form was developed by the P.C. to consolidate a range of variables (Box 2) through an iterative process. Data were then extracted and imported into a Microsoft Excel spreadsheet, which was summarised both numerically and by quantitative thematic analysis (stage 5). Study characteristics included socio-economic and cultural factors, university–community capacity and readiness, health issue importance; group dynamics and equitable partnerships; the type of intervention implemented during the project; and outcomes describing the desired effects, unwanted effects or side effects that arose.^12^

BOX 2 The variables charted from included literature on community-based participatory research in the prevention and control of hypertension.

**Variables charted from included literature**

- **Study context**
  ■ Socio-economic and cultural factors
  ■ University–community readiness
  ■ Study duration, importance of health issue of HT
- **Group dynamics and equitable partnerships**
  ■ Community-based partnerships
- **Type of intervention implemented during the project**
  ■ Study type or methodology
  ■ Aims of intervention
  ■ Health education platforms
  ■ Dissemination and utilisation of results or findings
  ■ The use of health behaviour changes modification theory
- **Outcomes**
  ■ Results of HT outcomes, prevalence or risk factors for documented HT
  ■ Capacity building or training of community members

HT, hypertension.

We collated numerical summaries for the type of method, study site, country of publication, duration of implementation, population description (number of participants) and duration of implementation. We also collated qualitative summaries for CBPR implementation, HT outcomes, behaviour change, capacity building, and the uses of evidence generated from CBPR implementation.

### Ethical consideration

The scoping study was part of a bigger project that had an ethical approval obtained jointly from the Medical Research Council of Zimbabwe (MRCZ/A/2136) and the Biomedical Research Council of the University of KwaZulu-Natal, South Africa (BFC318/16).

## Results

### Description of studies

**FIGURE 2:**
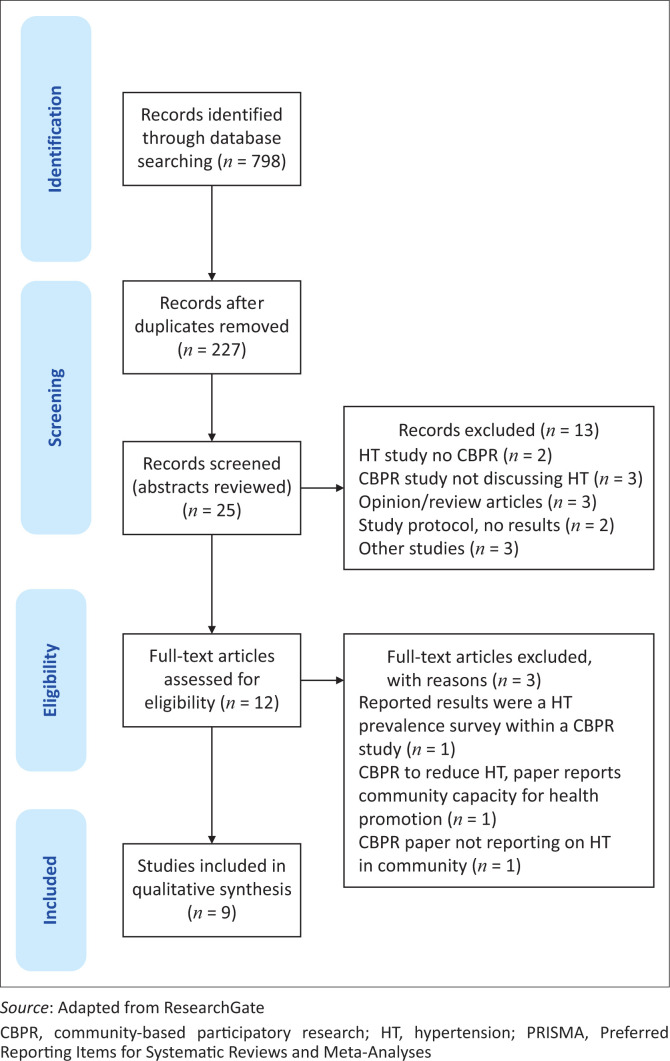
PRISMA flow diagram showing records that were retrieved and screened on the use of community-based participatory research in hypertension management.

Using the eligibility criteria and a peer-review process, nine studies were ultimately included in the analysis. Figure 2 shows the outcomes of the study identification and selection process.

### Study characteristics

Table 1 shows a summary of the study characteristics indicating citation, study context, group dynamics and outcomes.

**TABLE 1:** Study characteristics for studies using participatory action research in hypertension management.

Author or citation, country, publication year	Context (importance of health issue, socio-economic and cultural factors, community and university capacity and readiness)	Group dynamics (structural and individual dynamics) and intervention (community explanatory models, translation, implementation and dissemination; appropriate research design)	Outcomes: system and capacity changes (policies and practices, sustainable interventions, changes in power relations, cultural renewal); improved health (reduced disparities, increased social justice)
Skolarus et al.^18^	US facing a HT epidemic with 30% of adult population having the disease. The burden of HT is highest in African Americans (43%), compared to 28% of non-Hispanic whites. African Americans are likely to have their BP controlled and are at a higher risk. Underserved community with 60% African Americans. More than 40% live below the PDL.	The CBPR framework ‘reach-out’ used was a behavioural intervention that influenced health behaviour and was theory based, as well as being a faith collaboration. A randomised pilot interventional trial of four mobile health components to reduce BP among African Americans was conducted. Academic–community partnerships were formed with churches recruiting, delivering interventions and disseminating findings. A total of 425 church members were screened for HT; 94 were enrolled in the study and 73 (78%) completed the 6-month outcome assessment. An FGD with 38 African American adults was conducted between November 2011 and March 2012. A CBPR partnership was established in 2009 through the University of Michigan’s stroke programme, with community partners Bridges into the Future. Weekly communication of the partners on stroke prevention was held. Reach-Out is a faith-based collaborative, where recruitment and delivery of the intervention with the involvement of the church health team. Participants were recruited from three African American churches following church services. These were adults above the age of 18 years, who owned a phone and who had a BP of >140/90. They signed informed consent and were randomised into usual care or the intervention group. Self-determination theory provided the behavioural framework (competence, autonomy and relatedness), which contributed to intrinsic motivation and sustained behaviour change. Components of intervention included: BP self-monitoring, a tailored SMS for BP results, a health behaviour SMS and a generic health behaviour SMS. Written materials from the American Heart Association on the importance of HT among African Americans was made available. The assessments were done after 6 months.	The median age of participants was 58 years and 79% were women. The outcomes measured were: ease of recruitment of hypertensive patients, acceptance of randomisation, adequate study retention, high participant engagement, high acceptability of the programme, and the ability to target high-risk, vulnerable groups that may be missed in a normal medical clinic-based effort. Financial barriers to medication and adequate nutrition (food insecurity) were also measured. A total of 495 church members were screened and 94 participants enrolled in the study; 97% were African Americans and 79% were women. Stroke and heart attack were the most common consequence of HT (32% each) among intervention participants. Participants responded on 13.7 (SD = 10.7) weeks. Response to post intervention surveys and FGD (acceptance of intervention); the overall satisfaction was 100%; 84% found the BP monitor easy to use and 86% chose to continue receiving text messages. A total of 81% thought that the length of the intervention was just right. Eighty-five percent had a known family history of HT, 85% took medication with compliance and 85% were under the care of a service provider. On clinical outcomes, BP control: the within group systolic BP change was −11.3 (SD = 22.9) mmHg and within control group was −14.4 (SD≈= 26.4) mmHg. On participant characteristics, 20% had low health literacy, 30% had cost-related medication non-adherence. Food insecurity in the previous year was high, with 45% who could not afford enough food and 38% who could not afford a balanced diet. The FGD results indicated there was existing community awareness of a high prevalence of HT and interest in healthy lifestyle strategies. The need to address high BP among local FBOs was highlighted.
Baker et al.^19^	Cardiovascular diseases are the leading cause of death among African Americans in the US, with HT and obesity being the main contributing factors. Nutrition is the main predictor of obesity and HT. African Americans were less likely to adhere to DASH diets compared to whites, mainly due to the cost of food, food availability, culture and additional adaptability. The study was located in rural counties in the south western parts of Missouri, US. Pemiscot County had a population of 17 800, of which 26% were African Americans. A total of 55% of the African American population had no high school education and 56% lived below the PDL. Dunklin County, adjacent to Pemiscot County, had approximately 31 700 residents, of whom 10% were African Americans. Heart disease mortality rates in both counties was higher that the mortality rate in Missouri. There was a general consensus that increasing fruit and vegetable consumption and decreasing fat and salt intake are important. The DASH diets have been recommended as one way to improve dietary behaviours and decrease HT. To be effective, a nutritional intervention must address a combination of individual, cultural, social and environmental determinants.	The implementation of a CBPR project with partnership between academics, local community leaders, business people, mayors and a regional economic advisor, the aim of which was to change a particular behaviour in a community. Men on the Move: Growing Communities was the implementing organ in Pemiscot and Dunklin counties, Missouri, US. Surveys were done on rural African Americans >18 years prior to the intervention in 2008. The project ended in 2013 and a final analysis was done in 2015. The MOTMGC used the CBPR approach to provide culturally appropriate education and changes to the environment to improve access to fruit and vegetables, and low-fat and low-sodium foods. Health education on the REACH message was made available through flyers, family dinner nights and recipe tastings in the communities. A monthly newsletter with heart-healthy messages was printed and shared. The intervention was to adapt a dietary approach to stop HT. The DASH (or low-sodium) diet was recommended to improve dietary behaviours and decrease HT among the African American community. This included collaboration with community organisations and businesses to provide culturally appropriate environmental and dietary changes and improving access to fruit and vegetables as well as low-salt and low-fat foods. The MOTMGC used CBPR to address individual, environmental and social determinants of cardiovascular disease. The DASH and low-sodium diets were advocated to improve the chances of making heart-healthy choices. There were six community gardens whose produce was given directly to the community, sold or given to retail shops and restaurants. Community members were trained to manage the gardens. The local grocery stores agreed to carry low-salt and low-fat products. Health education practices were based on the social cognitive theory. Self-reported behaviours were measured using the trans-theoretical model. Activities and materials focused on the REACH message (reach for larger portions of fruits and vegetables, eat less salt, season vegetables with less fat), which was conducted by trained community health advocates. Blood pressure was measured, BMI calculated and behavioural risk factor surveillance done.	About 1200 individuals participated in one or more of the activities. There was a decline in the prevalence of HT, obesity and overweight respondents in the intervention, but not in the comparison county. There was a high participation (74%) of respondents in the intervention county who participated in the nutrition education programmes compared to 24% in the control county. The participants reported that due to access to MOTMGC nutrition gardens, they were more likely to eat fruit and vegetables, locally produced fresh foods and less processed fast foods. Those with high participation in MOTMGC had favourable healthy diets compared to those who did not participate. Adapting the DASH diet to community settings through culturally appropriate, community-based efforts can improve dietary behaviours, BMI and BP.
Liao et al.^20^	The primary focus was to address upstream health disparities in poverty-stricken minority Hispanics. Hispanics experienced poor health status in the US. Cardiovascular diseases were the leading cause of death in this community, with HT being a major risk factor. With the Hispanic community having a poor awareness of HT, they were less likely to be instructed by their physicians to take antihypertensive medication, to adopt lifestyle modifications to control BP or to follow medical advice once given. Research was conducted to work with racial and ethnic minority populations to eliminate health disparities.	A CBPR intervention called Racial and Ethnic Approaches to Community Health across the US (REACH U.S.) was conducted between 2009 and 2012 and funded by the Centers for Disease Control and Prevention. This was done in 40 communities with one or more ethnic or racial group. Health priorities were mostly NCDs (CVDs and diabetes mellitus). Six of these communities chose CVDs as the only one, or one of, the priority areas and these were reported in this study. Intervention had three major approaches: to build strong community-based coalitions; to focus on policy, systems and environment changes; and the cultural and linguistic tailoring of interventions. Community-based coalitions were made up of CBOs, health departments and universities and were primarily driven by residents. The coalition assessed disparities in healthcare access and outcomes, and advocated for equitable healthcare access and service delivery. The organisation REACH U.S. promoted access to healthy foods, and community and environmental changes with regard to food security. There was a promotion of culturally and linguistically appropriate messages tailored to target the population’s health literacy level. A risk factor survey was also undertaken. There was a 4-year follow-up period. A mixed methods study was conducted.	There were 968 hypertensive Hispanics who responded in 2009; the figure rose to 1455 in 2012. Significant improvements in medicine compliance, cutting down on salt and a reduction in alcohol use were reported among self-reported hypertensive Hispanics in REACH communities. There was a promotion of a healthy diet and physical activity. Interventions were culturally tailored. A CBPR intervention led to Hispanic residents in communities taking action to control HT. Clinical management, knowledge, beliefs and cultural competencies were improved.
Jones et al.^21^	Significant disparities exist among minority groups in relation to the prevalence of HT in Canada, especially among South Asian (SA) Canadians. A study was undertaken to determine the feasibility of implementing a sustainable, culturally adapted, community-based CVD risk-factor screening programme in places of worship for SA Canadians 45 years of age and older. South Asian Canadians are the second largest visible minority group in Canada. They suffer disproportionately high rates of CVDs 2–5 times higher than those of Chinese or European descent. South Asian Canadians have their first myocardial infarctions on average 5 years earlier than the general population in Canada and elsewhere. This due to the high prevalence of diabetes and metabolic syndromes with dyslipidemias. Additional contributors are language difficulties, low health literacy, decreased medicine adherence, disparate health beliefs, and a lack of knowledge, understanding and appreciation of the serious nature of CVDs.	Community-based participatory research carried out on minority groups in their own community with tailored interventions that target specific barriers have been shown to be effective in reducing disparities in care. Preferably faith-based interventions, carried out by CHWs or lay volunteers, with the use of health education materials specifically adapted for language, culture and literacy needs of the minority group, have shown promise to achieve better results. Religious facilities were chosen by community leaders as screening locations. Local family physicians, 49 SA Canadians lay volunteers, pharmacists, dieticians, nurses and medical students formed part of the team. They were trained on the validated culturally adapted volunteer trained tool. They were trained to assess CVD risk, provide health education and advice, referral to family physicians and to local culturally tailored CDM programmes. Baseline screening: SA Canadians (non-pregnant) were screened and programmes were presented in their preferred language of choice. Demographic and risk-related data were collected using questionnaires, and resting BP was measured. Those with identified risk were further studied to test for TC/HDL ratio and a calculation of 10 years CVD risk. Screening results and culturally adapted health education materials were given to participants; they were asked to follow up with their family physician within 1 month of screening. Follow-up screening was done after 6–13 months of consecutive first 100 participants. A total of 374 presented for screening; 238 participants were eligible and were screened between May and November 2015. Forty-nine SA Canadians lay volunteers were trained. Allied healthcare professionals (two nurses, two dieticians, two pharmacists and five medical students) were also trained as volunteers. Follow-up screening of participants was done after 6–13 months (median of 9 months) of the first consecutive 100 participants.	Ninety-nine participants attended screening. Forty-seven percent were female; 82% had access to healthcare providers, 22% reported medication changes and 3.2% had attended CDM programmes. While HT remained unchanged, TC and TC/HDL ratios reduced and HDL increased significantly. Thirty-six percent had elevated BP, 58% had elevated TC/HDL ratios, 23% reported DM and 76% had a high risk of CVDs. Those with DM were more likely to have HT. On follow-up there was a decrease in TC/HDL ratios. Mean (systolic and diastolic) BP levels did not change and remained elevated in 35% of the cases. On re-evaluation, 35% wanted to know their BP and cholesterol levels, 31% were following up a pre-existing condition, 58% were worried about the information they had received and 82% had visited their family physician. Eighty two percent had access to healthcare providers, 22% reported medication changes and 3.2% had attended CDM programmes. Low attendance of CDM was due to time constraints and a lack of perceived need. While BP remained unchanged, TC and TC/HDL reduced and HDL increased significantly. Participants were very satisfied (80%) or satisfied (20%) with the project. Participants suggested that screening programmes and CDM programmes be more accessible by delivering evening or weekend programmes at more sites, providing transport, offering multilingual programmes or translation services, reducing screening waiting times and increasing the number of project staff. The S-CHAMP demonstrated the feasibility and value of implementing lay volunteer-led, culturally adapted, sustainable, opportunistic CVD risk-factor screening projects in places of worship among minority ethnic groups. Community buy-in, ownership and the strong commitment of lay community members has led to the dissemination of the programme beyond its original settings. Places of worship play a cultural and social role in many communities and studies suggest they are feasible sites for identifying individuals with CVD risks.
Lucumí et al.^22^	Hypertension is a growing problem throughout Latin America, with one quarter of the adult population reported to have HT. Limited attention given to social and economic factors that determine the distribution of HT in disadvantaged urban areas contributes to the increased risk of HT. This was a marginalised urban, poverty-stricken community with a sizeable displaced community due to armed conflict. Marginalised urban areas of the LMIC community in Quibdo, Colombia, has the highest levels of poverty, at 50%. Displaced residents (about 20%) of the community were forcibly displaced from their former communities. Social determinants include forced displacement, unemployment, unplanned urban space, low social capital and a lack of facilities for physical activity. These were linked to stress, poor dietary practices and a lack of exercise. There was little attention given to CBPR to address the social determinants of HT. Constrained economic opportunities, poor physical infrastructure, reduced social cohesion, exposure to high crime levels and social maladies were prevalent. Living under these circumstances led to behavioural and psychosocial mechanisms that contributed to the increased risk of high BP. Membership of the coalition was varied from time to time depending on the degree of readiness and capacity for engagement.	The adoption of a framework of social determinants of HT to address complex health problems and iniquities requires multiple stakeholder participation. A coalition CHRG to address social determinants of HT in Quibdo was formed. In May 2013, the CRGH stakeholder analysis included community-based organisations, academia, the government (health, sport and recreation) and community organisations which represented displaced population representatives and social services. Twelve community organisations, government departments and academics formed a coalition and defined goals to advocate for health education and the implementation of evidenced-based, culturally acceptable innovations to reduce HT in the community. A general assembly of members was responsible for decision-making. The≈coalition worked to refine a vision and developed an action plan towards HT prevention and control. The study commenced in 2013 and had programmes continuing in 2018. A qualitative participatory action research study was carried out. The study design was discussed with stakeholders and with 12 organisations committing to participating in the study implementation. Core community organisations participated in making decisions. Priorities included an improved understanding of SDH from research, evidence-based and culturally sensitive interventions, and training of coalition members as part of capacity building. Participatory approaches used included the active and equitable engagement of all partners throughout the process. Components of the plan included a community survey on cardiovascular health and disease (December 2016) and workshops on strengthening capacity to conduct CBPR (September 2015 and June 2017). The dissemination of the community survey results was to inform the 2018 action plan.	There was successful coalition formation with defined goals and objectives that culminated in a shared vision. Capacity building in the form of community control in agenda-setting and decision-making was developed among the marginalised communities. There was improved engagement with the community, enhanced understanding of the problem, a strengthening of leadership, and the creation and maintenance of networks. The promotion of health and education was undertaken to improve community awareness on the social determinants of HT. However, there were no substantive data to back up the long-term effects of reducing HT that could be proven. There was a need to pay particular attention in marginalised LMIC by implementing coalitions to identify context-specific challenges and opportunities to enable reframing health and strengthening capacity. The SDH framework offered the opportunity of connecting socio-economic concerns to the increased risk of HT and other adverse health outcomes, with an opportunity to engage multiple stakeholders in planning for health promotion and equity. The importance of community control in decision-making to assure the active participation of marginalised communities was key.
López-Mateus et al.^23^	Increased life expectancy in Columbia was followed by a high prevalence of NCDs in the elderly, highlighting HT as the most prevalent disease in the territory. There was a need to conduct studies that would promote health in the elderly. Global adherence to chronic treatments is low at 50%; there were reported challenges of defaulting medicines at 22.4%, as reported by specialist doctors. The context of the research was to determine why older adults have a low adherence to healthy habits. There was a need to promote adherence to reduce the risk of complications. There was a need to implement strategies that improve a patient’s adherence to treatment and healthy living habits while approaching them in a holistic manner. There was a lack of educational programmes that integrated the cultural context, traditions and specific motivations of each community.	A CBPR qualitative study was done with elderly patients attending adult day-care centres, with 121 seniors aged between 60 and 90. A coalition was formed between the patients, health workers and university researchers. Power was shared equally between the researchers and the community. Work plans were developed on community diagnosis and problem identification, with the active participation of the community, followed by the codification of information. Strategies were tailored for elderly hypertensive patients, such as identifying resources and developing a health education strategy as part of the comprehensive management of HT. Knowledge about HT of adults and facilitators was explored, as well as their reasons for poor adherence to treatment. The community contributed to developments that promoted health in the elderly. Activities were in the form of traditional games and some were developed as homework for dissemination.	A total of 121 adults participated in this study, of which 64% were women. Of these, 65% were not natives of the municipality of Sopo but had lived for more than 20 years in the town. The majority lived with their children and grandchildren and were supported by their children, including a subsidy from the municipality. The five facilitators did not have HT but could relate to family experiences. An effective and sustainable intervention to control HT in the elderly was achieved by the appropriation of agricultural resources, encouraging dance as a form of exercise, the use of motivational strategies, the support of institutions that work with the elderly and empowering the facilitators. Resuming traditions and customs as a source of knowledge had to be done gradually as most respondents were not native to the area. Encouraging the elderly to take part in physical activity to maintain a healthy physical lifestyle was key, while motivating the community to stay healthy was also undertaken. Facilitators had a role in maintaining health among the elderly through themes of activities they developed and through training. This, combined with pharmacological management, resulted in the most effective control of HT.
Lin et al.^24^	There was a high burden and prevalence of HT among adults in China. Evidence from randomised control trials suggests that lifestyle interventions (e.g. weight loss, dietary modifications and increased physical activity) effectively lowered BP and glucose but are not offered in healthcare settings. Guidelines for the prevention and control of HT emphasise health education and the availability of lifestyle interventions through primary care services. There are 22 community health clinics – one for each neighbourhood. Research was conducted to evaluate the effectiveness of a community-based lifestyle intervention on BP control in a middle-aged and older Chinese population.	A community-based lifestyle intervention study was conducted to determine whether an intervention delivered by field health workers for middle-aged and older adults in an urban, resource-limited community could be effective in reducing BP and glucose, and whether any beneficial effects could be sustained in the long-term. There was collaboration between local residential committees, a community health team and a research team of academics. A cluster randomised control trial was carried out with an intervention group: A 12-month lifestyle promotion programme was administered through an existing community-based system for the management of HT and DM. The framework was based on the health belief model, which hypothesises that a particular form of behaviour depends on the individual personal beliefs about the perceived threats posed by a health problem. Training involved a demonstration on healthy dietary patterns, food preparation methods, participating in regular physical exercise, and resistance and relapse prevention skills for smoking and alcohol use. Participants were encouraged to adopt a healthy lifestyle, to consume culturally acceptable and economically feasible foods and to take part in a customised physical exercise regime. Reinforcement and support was offered for participants’ self-efficacy in maintaining a healthy lifestyle and emphasising long-term adherence. Patients with HT were given tailored healthcare. The control group received conventional health education on common chronic diseases. A total of 474 participants 50–79 years of age were assigned to intensive health education and behavioural intervention, or to the control group that received conventional education. A routine physical examination was done and information on lifestyle changes was collected. Participants were followed up at 6, 12 and 24 months. The follow-up period took place between January 2010 and March 2012.	At 12 months follow-up in the intervention group there was a significant reduction in systolic BP (−4.9 mmHg vs 2.4 mmHg MD 7.3 mmHg; p < 0.001) and diastolic BP (−1.9 mmHg vs 1.9 mmHg MD −3.8 mmHg; p < 0.001) and fasting blood glucose (−0.59 mmol/L vs 0.08 mmol/L; MD 0.67 mmol/L; p < 0.001). These differences were sustained at the 24-month follow-up and the intervention group reported a sustainable decrease in the self-reported intake of salt and cooking oil at the follow-up period. The intervention group also reported a significant increase in vegetable intake at follow-up. Participants in the intervention increased their activity levels one to two-fold during the follow-up period. Medication use improved among both groups although those in the intervention group were more likely to adhere to their pharmacological treatment. This approach of lifestyle interventions conducted through primary care services may be a potential solution for combating HT and diabetes in resource-limited settings. The results indicated that comprehensive lifestyle interventions can be implemented effectively using a community-based approach, thus achieving long-term improvements in BP and glucose in a middle-aged and older population. Individuals who received intervention sustained behavioural changes and weight loss over the subsequent 12 months. A reduction in BP and glucose and the adoption of a long-term healthy lifestyle were associated with a reduced risk of CVD and mortality. The delivery of lifestyle intervention by community health staff is a promising vehicle for the primary prevention of CVD. Despite considerable effectiveness, the delivery of such intensive lifestyle interventions requires the involvement of community organisations, expertise, capacity development and resources. Several lifestyle changes including dietary intake, physical exercise and medication adherence responded favourably to the intervention.
Bradley et al.^25^	There is a generalised increase in the prevalence of CVDs in the black and disadvantaged populations in South Africa. Changes in dietary consumption along with a decrease in physical activity and other environmental factors have contributed to obesity in this population. Khayelitsha Site C is a disadvantaged urban area in Cape Town, South Africa, inhabited mainly by people who migrated from rural areas. Poverty and severe socio-economic health indicators were noted in the community. A total of 36% were employed, with 80% living in poor housing conditions. Social determinants of health were a lack of exercise and a lack of the promotion of healthy lifestyles in the community. Community health workers who participated in the study provided primary healthcare in the community. They lived in the area and shared the same socio-cultural and demographic profiles as the members of this community. The study aim was to identify factors that contribute to HT (and DM), to design and implement appropriate local interventions for prevention, and to promote healthy lifestyles.	A project on the primary prevention of HT and DM was carried out. A coalition was formed by CHWs, health workers and university academics. The coalition aimed at empowering the CHWs in the primary prevention of HT. There was community involvement in all phases of the research. A mixed methods study using both quantitative and qualitative methods was employed. The CHWs participated in the assessment, analysis and action taken. Using CBPR to implement programmes enabled active participation, the harnessing of skills and expertise of partners in bridging cultural gaps and engaging local knowledge. There was an assessment of CHWs’ knowledge, beliefs and attitudes. The cooking practices and eating patterns of CHWs were also examined. Interviews, questionnaires, FGDs and anthropometric measurements were used for data collection. All CHWs were taught to take anthropometric measurements. Community health workers were provided with chicken and maize and asked to demonstrate preparing and serving food for their families. Businesses and community mapping was carried out by CHWs and their neighbours on aspects contributing positively or negatively to their health. The analysis and interpretation of the results were done together and the presentation (dissemination) was carried out in a meeting with all the stakeholders, followed by a group discussion of the results. Implementation included the development of a training programme on the prevention of HT: improving knowledge; and promoting healthy lifestyles, good nutrition and physical activity including skills development in communication and advocacy. Individual interviews were conducted with all 42 CHWs. Two more focused group discussions (FGDs) were conducted with 27 CHWs. Two FGDs were undertaken with 17 conveniently selected CHWs. A health club was started by the CHWs. Six CHWs attended training sessions with sports scientists on how to lead exercises. Members who joined the health club discussed issues related to nutrition, a healthy diet and food preparation after the exercise class. Members were screened for BP monthly and referred to the clinic where appropriate. The follow-up period was from 2000 to 2005.	Forty-two CHWs participated in the CHW assessment from Site B and Site C. Findings elicited a lack of knowledge among the CHWs and the community on HT and DM and the risk factors thereof. Economic constraints and cultural beliefs and practices influenced the community’s food choices and participation in physical activity. Poor individual knowledge was noted. Social and cultural contexts on food and body size and environmental factors had an influence on the prevention and control of HT in this community. For example, a large female body size was regarded as desirable in this community. Other factors such as economic constraints, limited food choices, long distances, high transport costs to the nearest supermarket and a lack of opportunities to engage in physical exercise were highlighted. The project was successful due to active participation, skills development and the community being empowered. Community-monitoring mechanisms were established. Community health-based care was linked to primary healthcare to enable the sustainability of service delivery. In addition, culturally appropriate prevention and treatment interventions were established. Health education sessions were held, as well as BP screening and appropriate referrals from the health club. The interventions were appropriate and sustainable in this community. About 200–250 community members attended screening and awareness activities and role plays over the course of 3 years. The health club had six trained CHWs with sports scientists and 30 members joined.
Chimberengwa et al.^26^	The study was located in a disadvantaged rural area in southern Zimbabwe where poverty and recurrent droughts were prevalent. It was a community where the prevalence of HT was estimated to be 26%. The community had difficulties with the availability of medicines and long walking distances to the health facility.	Health services research was conducted using community-based participatory action research. A CIG with 22 participants was formed, made up of hypertensive patients, CHWs, community leaders, nurses and a principal investigator. The CIG conducted a mixed methods study in phase one where activities focused on the primary, secondary and tertiary prevention of HT. There were monthly action reflection cycles, FGDs and in-depth interviews to collect data. The CIG made use of the WHO chronic care model to implement strategies on the primary prevention of HT. The project continued for 8 months – from April to November 2017. The CIG designed and carried out a CBPR on HT management. The CIG was trained and collected information on knowledge, attitudes and practices on HT by the patients in the community. Themes were developed from the quantitative data and, using action reflection cycles, programmes were developed and implemented in the community to improve the primary prevention of HT. This included a HT CIG club, HT days and outreaches. The CHWs were empowered to screen, diagnose and manage HT in the community.	Six CIG action reflection meetings were held. One visitor attended the first meeting, increasing to 30 by the sixth meeting. Hypertensive patients seen on hypertensive clinic days increased from 10 to 61. The eight hypertensive CIG members had their BP pressure well controlled by the end of the project. Forty-three new hypertensive patients were diagnosed from the community. Ten CHWs were trained on the use of digital BP monitoring, community diagnosis and patient monitoring. Hypertension registers were established and a combined total of 195 hypertensive-registered patients were handed over to the clinic at the end of the project. The CIG members were empowered; and patients had their BP controlled, developing faith in the HT service delivery package. The CHWs were empowered with diagnostic and management competencies on HT care, while the community developed trust in the CHWs and the clinic system. Myths and misconceptions about HT in the community were corrected through the project’s activities.

HT, hypertension; CBPR, community-based participatory research; BP, blood pressure; PDL, poverty datum line, FGD, focused group discussion; FBOs, faith-based organisations; US, United States; DASH, Dietary Approaches to Stop Hypertension; MOTMGC, Men on the Move: Growing Communities; REACH, Racial and Ethnic Approaches to Community Health; BMI, body mass index; NCDs, noncommunicable diseases; CVDs, cardiovascular diseases; CBOs, community-based organisations; DM, diabetes mellitus; S-CHAMP, South Asian Cardiovascular Health Assessment and Management Program; LMICs, low- and middle-income countries; CDM, chronic disease management, TC, total cholesterol; HDL, high density lipoprotein; CHRG, community health research group; SDH, social determinants of health; CI, confidence interval.

### Study context

Three of the reviewed articles were written in the United States (US),^18,19,20^ one in Canada,^21^ two in Colombia,^22,23^ one in China,^24^ one in South Africa^25^ and one in Zimbabwe.^26^ Only one study had been published before the year 2010,^25^ while the rest were published between the years 2010 and 2020.^18,19,20,21,22,23,24,26^ The study setting was predominantly in an urban locale (*n* = 6),^18,20,21,22,23,24^ with the other three studies having been conducted in a rural setting.^19,25,26^ All of the studies were conducted in poverty-stricken communities that were characterised by constrained economic opportunities, poor physical infrastructure and reduced social cohesion, all contributing to a higher prevalence of HT and ill health. In Africa,^25,26^ studies conducted were undertaken in black communities. In Europe, studies were on minority ethnic or racial groups which included African Americans,^18,19^ Hispanics^20^ and South Asian Canadians.^21^ Two studies were conducted in Colombia; the first one was on displaced marginalised communities,^22^ and the second was on the secondary and tertiary prevention of HT in the elderly.^23^

The importance of HT as a key contributor to cardiovascular diseases (CVDs) was emphasised by all the studies.^18,19,20,21,22,23,24,25,26^ The social determinants of the community in question were described by all of the studies (*n* = 9),^18,19,20,21,22,23,24,25,26^ which included socio-economic, cultural and environmental factors. All of the studies were undertaken to address health disparities among different groups of people in the community, thus the aims of the study were stated.^18,19,20,21,22,23,24,25,26^ The number of study participants was stated in eight of the reviewed articles; two had a total number of participants of below 200^23,25^; four had participants of between 200 and 500^18,21,24,25^; and two had participants of more than 500.^19,20^ The studies covered a wide range of HT prevention strategies – primary,^19,22,25,26^ secondary^18,19,21,25,26^ and tertiary prevention.^14,15,16,17,19,20,21,22^

### Group dynamics and equitable partnerships

All of the reviewed articles described the formation of a coalition that included the community and the academics or researchers.^18,19,20,21,22,23,24,25,26^ The focus was to address complex health problems and inequities utilising multiple stakeholder participation. Various coalitions were formed and given special names; for example, ‘community health research group’ (CHRG),^22^ ‘Racial and Ethnic Approaches to Community Health across the US’ (REACH U.S.),^20^ ‘Men on the Move: Growing Communities’ (MOTMGC)^19^ and ‘cooperative inquiry group’ (CIG).^26^ Other coalitions were not given specific names.^17,19,20,21^ All of the partnerships included university departments or academics and community organisations, while health workers and government departments were incorporated depending on the type of study and setting. Community organisations included community-based organisations (CBOs), non-governmental organisations (NGOs), faith-based organisations (FBOs) and the business sector.

All of the studies adopted the social determinants of health (SDH) to address health disparities,^18,19,20,21,22,23,24,25,26^ looking at various perspectives such as displacement,^18^ racial and ethnic minorities,^18,20,21^ senior citizens in the society^23,24^ and other vulnerable black communities.^15,21,22^ There was equitable power-sharing between the academic–community partnerships. There was active participation in decision-making by the community members in determining the focus, implementation and dissemination of findings in all the studies. The importance of community control in decision-making ensured the active participation of marginalised communities. The use of CBPR partnerships to implement programmes enabled active participation, harnessing skills and expertise of partners in bridging cultural gaps and engaging local knowledge.^18,19,20,21,22,23,24,25^ In the process, these partnerships promoted the active and equitable engagement of all partners.

### Intervention

Various methodologies were utilised in implementing CBPR, with all studies^18,19,20,21,22,23,24,25,26^ having reported the use of CBPR interventions in managing HT. In the implementation of CBPR, the following characteristics were described in all the studies (*n* = 9): (1) a description of research participants, (2) the ages of research participants and (3) the aims and objectives of the intervention.^18,19,20,21,22,23,24,25,26^ Two studies used qualitative methods,^22,23^ where the community research group on health (CRGH) conducted a study on innovations on the primary and secondary prevention of HT in the community,^22^ while in the other study the coalition was working on the tertiary prevention of HT in elderly hypertensive citizens.^23^

Three studies reported having used mixed methods (qualitative and quantitative).^20,25,26^ The REACH U.S. study focused on CVDs as a priority in communities. The intervention had three major approaches: to build strong community-based coalitions; to focus on policy, systems and environment (PSE) changes; and the cultural and linguistic tailoring of interventions.^20^ A study on the primary prevention of HT was carried out where a coalition was formed to enable village health workers (VHWs) to actively participate in harnessing the skills and expertise of community members in order to bridge cultural gaps and engage local knowledge.^25^ The third study utilised a CIG to implement measures focused on the primary, secondary and tertiary prevention of HT in the community.^26^

Two studies were quasi-experimental CBPR interventions.^19,21^ The MOTMGC used CBPR to address individual, environmental and social determinants of CVD by advocating for Dietary Approaches to Stop Hypertension (DASH) and low-sodium diets, improving the possibility of people making heart-healthy choices.^19^ The aim was to change a particular behaviour in a community, with MOTMGC being the implementing organ to provide culturally appropriate education and changes to the environment to improve access to fruits and vegetables, and low-fat and low-sodium foods. The second quasi-experimental study reported CBPR carried out on minority groups with tailored interventions that targeted specific barriers; it was shown to be effective in reducing disparities in care and that making use of faith-based interventions was helpful.^21^ This research was carried out by community health workers (CHWs) or lay volunteers with the use of health education materials specifically adapted for the language, culture and literacy needs of the minority groups. Religious facilities chosen by community leaders were used as screening and follow-up locations.

Two studies were conducted using randomised control trials (RCTs).^18,24^ The CBPR framework ‘reach-out’ used was a behavioural intervention that influenced health behaviour and was theory based, as well as being a faith collaboration. A randomised, pilot interventional trial of four mobile health components to reduce blood pressure (BP) among African Americans.^18^ A cluster randomised control trial was undertaken to determine whether an intervention delivered by field health workers for middle-aged and older adults in an urban resource-limited community could be effective in reducing BP and glucose, and whether any beneficial effects could be sustained in the long-term.^24^ The intervention group was exposed to a 12-month lifestyle promotion programme administered through an existing community-based system for the management of HT.

The follow-up period after the implementation of CBPR was stated in all of the reviewed articles (*n* = 9), which varied from a minimum of 6 months to a maximum of 5 years. Three studies took less than a year to complete,^18,21,26^ one study took 2 years to complete,^24^ and five studies took between 4 and 5 years to complete.^19,20,22,23,25^ All of the articles (*n* = 9) had aims and objectives of the CBPR intervention clearly stated and (*n* = 8) of the studies^18,19,20,21,22,24,25,26^ had indicators that were being traced throughout the follow-up period. All of the studies (*n* = 9) recorded specific parameters and key events before and after the implementation process to measure changes that were attributable to the intervention. The dissemination and utilisation of the findings were reportedly done by the community members in the coalition.^20,22,23,25,26^

All of the studies had a primary objective of influencing human behaviour. To hypothesise changes in human behaviour, three studies provided a behavioural framework: the self-determination theory,^18^ the trans-theoretical model^19^ and the health belief model (HBM).^24^ The rest of the studies,^20,21,22,23,25,26^ although they did not state the theory or model that determines changes in human behaviour, hypothesise that the social determinants in the community affect human behaviour. To achieve a change in behaviour while implementing CBPR, various health education platforms were created and reported in all of the studies.^18,19,20,21,22,23,24,25,26^ Health and education platforms were promoted in order to improve community awareness on the social determinants of HT.^18,19,20,21,22,23,24,25,26^ In all of the studies, the interventions were tailored to be culturally and linguistically appropriate.^18,19,20,21,22,23,24,25,26^

## Outcomes

### System and capacity changes

The studies reported detailed quantitative clinical outcomes from the intervention.^18,21,24^ In the ‘reach-out’ study, the median age of participants was 58 years and 79% were women.^18^ A total of 495 church members were screened and 94 participants enrolled in the study. Stroke and heart attack were the most common consequences of HT (32% each) among intervention participants.^18^ In terms of BP control, the within group systolic BP change was −11.3 (standard deviation [SD] 22.9) mmHg and within control group was −14.4 (SD = 26.4) mmHg. In the quasi-experimental study, 99 participants attended screening: 47% were female, 82% had access to healthcare providers, 22% reported medication changes and 3.2% had attended chronic disease management (CDM) programmes. Total cholesterol (TC) and TC/high density lipoprotein (HDL) ratios reduced and HDL increased significantly. Thirty six percent had elevated BP, 58% had elevated TC/HDL ratios and 76% had a high risk of CVDs. On follow-up there was a decrease in TC/HDL ratios.^21^ The third study on community-based lifestyle intervention reported that at the 12-month follow-up in the intervention group, there was a significant reduction in systolic BP (−4.9 mmHg vs 2.4 mmHg mean difference [MD] 7.3 mmHg; *p* < 0.001), diastolic BP (−1.9 mmHg vs 1.9 mmHg MD −3.8 mmHg; *p* < 0.001) and fasting blood glucose (−0.59 mmol/L vs 0.08 mmol/L; MD 0.67 mmol/L; *p* < 0.001). These differences were sustained at the 24-month follow-up and the intervention group reported a sustainable decrease in self-reported intake of salt and cooking oil at the follow-up period.^24^

There was successful coalition formation with defined goals and objectives that culminated in a shared vision in all the studies.^18,19,20,21,22,23,24,25,26^ The health promotion and health education was carried out to improve community awareness on social determinants of HT in all the studies.^18,19,20,21,22,23,24,25,26^ Resuming traditions and customs, including culturally tailored interventions, was specifically mentioned in four studies.^21,23,25,26^ An effective and sustainable intervention to control HT in the elderly was achieved by the appropriation of agricultural resources, encouraging dance as a form of exercise, the use of motivational strategies, supporting institutions that work with the elderly and empowering facilitators.^23^ Social and cultural contexts on food, body size and environmental factors had an influence on the prevention and control of HT in this community. For example, a large female body size was regarded as desirable in this community.^25^ Another study reportedly demonstrated the feasibility and value of implementing lay volunteer-led, culturally adapted sustainable opportunistic CVD risk factor screening in places of worship among minority ethnic groups. Where places of worship play a cultural and social role in many communities, studies suggest that they are feasible areas to identify individuals with CVD risk.^21^

Where lifestyles were improved to be more healthy, including reinforcing physical activity and reducing salt in the diet, healthy eating was reported.^19,20,24,25,26^ Significant improvements in medicine compliance, cutting down on salt and a reduction in alcohol use were reported among self-reported hypertensive Hispanics in REACH communities.^20^ Also notable were improvements in clinical management, knowledge, beliefs and cultural competencies.^20^ Another study reported that the results indicated that comprehensive lifestyle interventions can be implemented effectively using a community-based approach, thus achieving long-term improvements in BP and glucose in the middle-aged and older population.^24^ Adapting the DASH diet to community settings through culturally appropriate, community-based efforts improved dietary behaviours, body mass index (BMI) and BP.^19^ The participants reported that as a result of access to MOTMGC nutrition gardens, they were more likely to eat fruit and vegetables, locally produced fresh foods and less processed fast foods. Those with a high participation in MOTMGC had favourable and healthy diets compared to those who did not participate.^15^

Capacity building and the empowerment of the community through coalitions took place, leading to the acceptability and sustainability of the interventions in all of the studies.^18,19,20,21,22,23,24,25,26^ Capacity building in the form of community control in agenda-setting and decision-making was developed among all the communities that were studied. There was improved engagement with the community, an enhanced understanding of problems, a strengthening of the leadership, and the creation and maintenance of networks. The SDH framework offered the opportunity to connect socio-economic concerns to the increased risk of HT and other adverse health outcomes, providing an opportunity to engage multiple stakeholders in planning for the promotion of health and equity.^18^ The interventions were appropriate and sustainable in the communities.^18,19,20,21,22,23,24,25,26^ Community buy-in, ownership and the strong commitment of lay community members led to the dissemination of programmes beyond their original settings.^18,19,20,21,22,23,24,25,26^ The CHWs were empowered with diagnostic and management competencies on HT care, and the community developed trust in CHWs and the clinic system.^26^ Five of the studies^20,22,23,25,26^ reported that the study findings were disseminated and utilised, including influencing key policymakers for health. All studies (*n* = 9) reported that there was evidence-based data that were generated to inform policy formulation.

### Improved health

The projects were successful due to active participation, skills development and the empowerment of communities.^18,19,20,21,22,23,24,25,26^ In one study, there was the establishment of community monitoring mechanisms and linked community health-based care to primary health-care to enable the sustainability of service delivery.^25^ The participation of community organisations such as local FBOs^18^ improved community awareness, as well as the implementation and dissemination of findings. Chronic disease management programmes were more accessible and this greatly improved health through educational programmes implemented in the community.^21^

Significant increases were reported in vegetable intake and compliance to medicines at follow-up, where participants in the intervention increased their activity levels one to two-fold during the follow-up period.^24^ This approach of lifestyle interventions conducted through primary care services may be a potential solution to combating HT in resource-limited settings.^24^ There was a documented decline in the prevalence of HT, obesity and overweight respondents in the intervention, but not the comparison county.^19^ All individuals who received intervention sustained behavioural changes.^18,19,20,21,22,23,24,25,26^

## Discussion

The findings from this scoping study confirmed that the use of CBPR in the prevention and control of HT was still an emerging field, as evidenced by the small number of studies that were found in the literature. The majority of the studies were published after the year 2010, with only one having been published prior to 2010.

Regardless of the context setting – whether rural or urban – the studies were conducted in disadvantaged communities where there was poverty, a lack of social cohesion and marginalisation. These communities included displaced, elderly hypertensive citizens, African Americans, South Asian Canadians and Hispanics. All of these groups were racial or ethnic minorities that experienced health disparities and lacked social justice. In all of the studies,^18,19,20,21,22,23,24,25,26^ the context of the study related to a high prevalence of HT. Socio-economic and environmental factors influenced the SDH. In all of the studies, the importance of HT as a key contributor to CVDs was emphasised.

The common finding was that for all of the studies,^18,19,20,21,22,23,24,25,26^ the context was that the communities were marginalised, had a high prevalence of HT and poor SDH. A CBPR approach (1) took into consideration context-specific challenges in marginalised communities,^18,19,20,21,22,23,24,25,26^ (2) aimed to provide culturally appropriate and logistically sound research by shaping the scope of the research, interpretation and dissemination of findings,^18,19,20,22,23,24,25,26^ (3) generated capacity to recruit community members to advisory boards and implementation by community members,^19,21,22,23,26^ (4) offered capacity for partnerships by researchers and the community^18,19,20,21,22,23,24,25,26^ and (5) offered long-term capacity to sustain the project goals beyond funded timelines.^18,19,21,23,24,25,26^

All of the reviewed articles described the formation of coalitions that included the community and the researchers. This offers the benefit of sharing ideas, including indigenous knowledge, while the academics could understand the social determinants of health from the community members themselves. Other community partners were also roped in to identify and address complex health problems. Moreover, the inequities were dealt with utilising multiple stakeholder participation. This process created a skills transfer within the community that participated in the project. There was community acceptance and ownership of the project, leading to its sustainability beyond project timelines. Even though differences were noted in the implementation of CBPR partnerships, the overarching principles of coalition formation were adhered to, including power that was shared equally, and active participation in all of the stages of the projects (as well as the dissemination of the results). Various methods were utilised for CBPR implementation and there was no standardisation of methods used, as seen with the use of qualitative mixed methods, quasi-experimental and RCTs in the studies.

The intervention had three major approaches: to build strong community-based coalitions; to focus on policy, systems and environment (PSE) changes; and the cultural and linguistic tailoring of interventions. All of the interventions which focused on minority groups had tailored interventions that targeted specific barriers that had been shown to be effective in reducing health disparities.

All of the studies had the primary objective of influencing human behaviour. Health behaviour modification theories were used to model CBPR to influence human behaviour. To achieve this change while implementing CBPR, various health education platforms were created and reported in all of the studies. Changes reported in HT outputs were as a result of a change in behaviour. To ensure behaviour change, community participation was encouraged through partnerships or the formation of coalitions. The application of CBPR principles of equitable power distribution and community participation with a shared vision contributed to favourable HT outcomes in the community. During project implementation, the community helped to shape the scope of the research, data collection, and interpretation and dissemination of findings. Socio-cultural contexts, traditions and hobbies of the community members affected by HT were taken into consideration when designing programmes for management that complement pharmacological therapy.

Partnerships or coalitions were used consistently across all CBPR implementation studies that were reviewed. The formation of coalitions was guided by well-defined goals and objectives where the inclusive team shared a common vision.^27^ Synergies built on success enhanced future successes in the community management of HT. The aim of the partnerships was to build capacity among inhabitants of disadvantaged communities living with a high prevalence of HT. In forming these partnerships, engagement occurred on equal terms between academics and community members. The ultimate aim of these coalitions was capacity building, which led to the empowerment of the community. Active participation brought about context-specific problem identification and measured task-shifting, and encouraged task-sharing and knowledge transfer which influenced behaviour modification or behaviour change.^27^ In addition, there was participant satisfaction and intervention sustainability in the community. This guaranteed improved community knowledge in the monitoring and control of HT.

The implementation of CBPR in turn improved clinical practice while addressing issues that were important to the community. Traditional types of research discouraged community experts from sharing invaluable expertise on perspectives and ideas as it did not foster community engagement and involvement.^28^ It has been noted that a few successful interventions often disappeared with the cessation of donor funding; therefore, CBPR offers a new approach which guarantees sustainable social change.^27^ The benefits of CBPR include capacity building and empowerment, and guarantees the sustainability of the intervention in the community post project funding timelines.

Healthy lifestyle improvement included reinforcing physical activity and reducing salt in the diet, and healthy eating was reported.^19,20,24,25,26^ Significant improvements in medicine compliance, cutting down on salt and the reduction in alcohol use was reported among self-reported hypertensive Hispanics in REACH communities.^20^ In another study, the primary prevention of HT was enhanced by encouraging a healthy diet such as the consumption of fruit and vegetables, locally produced fresh foods and less processed fast foods.^19^ This approach of lifestyle interventions conducted through primary care services may be a potential solution to combating HT in resource-limited settings.^24^ The projects were successful as a result of active participation, skills development and the empowerment of communities.^18,19,20,21,22,23,24,25,26^ All individuals who received intervention sustained behavioural changes.^18,19,20,21,22,23,24,25,26^ The delivery of lifestyle interventions by community health staff is a promising vehicle for the primary prevention of CVD.^24^ Ultimately, the implementation of CBPR improved health.

Despite a large body of research on HT documenting socio-economic disparities, access to health and quality of healthcare, appropriate interventions to improve health have lagged behind.^11,29^ By implementing CBPR, locally feasible, acceptable and sustainable strategies can be developed to address these key social determinants to health. A systematic review of randomised control trials to determine interventions used to control BP in patients conducted by Glynn et al. (2010) concluded that education alone, either to health professionals or patients, was not associated with a large net reduction in BP.^30^ There is a knowledge gap in the literature on the impact of CBPR on HT outcomes, and the available studies on the use of CBPR in cardiovascular medicine primarily focus on the prevention and control of HT. There were also challenges in determining the extent to which the effect of CBPR was noted in health disparities post intervention. This was because there is no scientific methodology to prove the actual impact of CBPR, as the method only infers the favourable outputs to the intervention.

The variations in methodologies to implement the CBPR intervention may have led to non-standardisation, hence the challenges that were reported in the studies. Similarly, there were no substantive data to back up the long-term impact of reducing HT that could be proved using CBPR methodologies.^22^ Some of the studies relied on the self-reporting of patients and this could have introduced bias into those studies.^20,22^ It was notable that economic constraints and cultural beliefs and practices influenced a community’s food choices and participation in physical activities.^25^ This became a challenge as these factors determined the primary prevention of CVDs. Despite considerable effectiveness, the delivery of such intensive lifestyle interventions to effectively contribute to behavioural change required the involvement of community organisations, expertise, capacity development and resources.^24^

## Conclusion and implications for further research

A CBPR framework can be used to define the context, group dynamics, implementation and outcomes of HT by using theories of human behaviour. The formation of academic–community coalitions help to address complex health problems and inequities utilising multiple stakeholder participation. The coalitions in CBPR aim to encourage community control in decision-making and ensure active participation throughout the project phases, including the dissemination of findings. Equitable power-sharing and engagement between the academic–community partnerships is promoted.

It is possible to apply the principles of CBPR in the primary and secondary prevention even though different methodologies can be used in the process. The aim of implementing CBPR is to change a particular behaviour in a community and to also address health inequities and disparities among minority groups in society. To achieve this, health education platforms specifically adapted for the language, culture and literacy needs of the minority groups should be implemented. In so doing, there should be a deliberate effort to build capacity and empower minorities. For primary and secondary prevention to be a success, an improvement to a healthy lifestyle needed to include reinforcing physical activity and reducing salt in the diet. Healthy eating could indeed be achieved through CBPR on HT. When properly implemented, CBPR will lead to the acceptability and sustainability of HT management and intervention strategies. The study findings from CBPR can be utilised to influence key policy changes for health planners.
